# OLDER ADULTS’ RESILIENT IDENTITY APPRAISALS: WHO AM I COMPARED TO MYSELF AND OTHERS?

**DOI:** 10.1093/geroni/igac059.2399

**Published:** 2022-12-20

**Authors:** Lauren Bouchard, Lydia Manning

**Affiliations:** Portland State University , Portland , Oregon, United States; Dele Health Tech, Naperville, Illinois, United States

## Abstract

Resilience is the adaptation of bouncing back from adverse life events. Older adults provide rich and varied resilience narratives of personal growth across the life course, especially related to others. Many people describe resilience as part of their identity, formed in childhood and maintained throughout adulthood. As adults age, they continually integrate new information into their identity, and aging presents long-term adversity and new life course challenges that older people must navigate. This qualitative study explored how older adults describe their sense of resilient identity compared to their younger selves and other older adults in their lives. Our narrative findings indicate resilience narratives often include comparative appraisals to the strengths and weaknesses of younger selves (e.g., “I am more emotionally resilient, but not as physically strong as I used to be”). Additionally, our findings show older adults comparing their resilience to others in a similar age category (e.g., “I do not complain like my friends”). Comparisons to other older adults fit two themes: empathic and connected and judgmental and distanced. We conclude the participants who view other older adults in an empathic and connected way maintained solid social connections and a general sense of interconnectedness, while those in the judgmental and distanced category might be reacting to internalized ageism. The social implications of these findings will be discussed in detail. In addition to specific examples, our study also provides limitations and future directions in resilient identity and resilience appraisals.

